# (3*R*,4*S*)-3,4,8-Trihy­droxy-1,2,3,4-tetra­hydro­naphthalen-1-one monohydrate from *Embellisia eureka*

**DOI:** 10.1107/S1600536812022623

**Published:** 2012-05-26

**Authors:** Tarik Ouchbani, Hafid Zouihri, El Mokhtar Essassi, Peter Proksch, Seik Weng Ng

**Affiliations:** a Laboratoire de Chimie Organique Hétérocyclique, Pôle de Compétences Pharmacochimie, Université Mohammed V-Agdal, BP 1014 Avenue Ibn Batout, Rabat, Morocco; b CNRST Division UATRS, Angle Allal Fassi/ FAR, BP 8027 Hay Riad, Rabat, Morocco; c Institut für Pharmazeutische Biologie und Biotechnologie, Heinrich Heine University Düsseldorf, Gebäude 26.23, Universitätsstrasse 1, 40225 Düsseldorf, Germany; d Department of Chemistry, University of Malaya, 50603 Kuala Lumpur, Malaysia; e Chemistry Department, King Abdulaziz University, PO Box 80203 Jeddah, Saudi Arabia

## Abstract

In the title hydrate, C_10_H_10_O_4_·H_2_O, the six-membered aliphatic ring that is fused to the benzene ring has a sofa shape, with the hy­droxy group in the 3-position (that represents the sofa back) of the aliphatic ring occupying a quasi-axial position. The hy­droxy group of the aromatic ring is hydrogen-bond donor to the carbonyl O atom; other O—H⋯O hydrogen bonds link the organic mol­ecules and the water mol­ecules into a three-dimensional network.

## Related literature

For the isolation of the title compound from other fungi, see: Borgschulte *et al.* (1991 ▶); Iwasaki *et al.* (1972 ▶); Trisuwan *et al.* (2008 ▶). The absolute configuration was assumed from published assignments, see: Trisuwan *et al.* (2008 ▶).
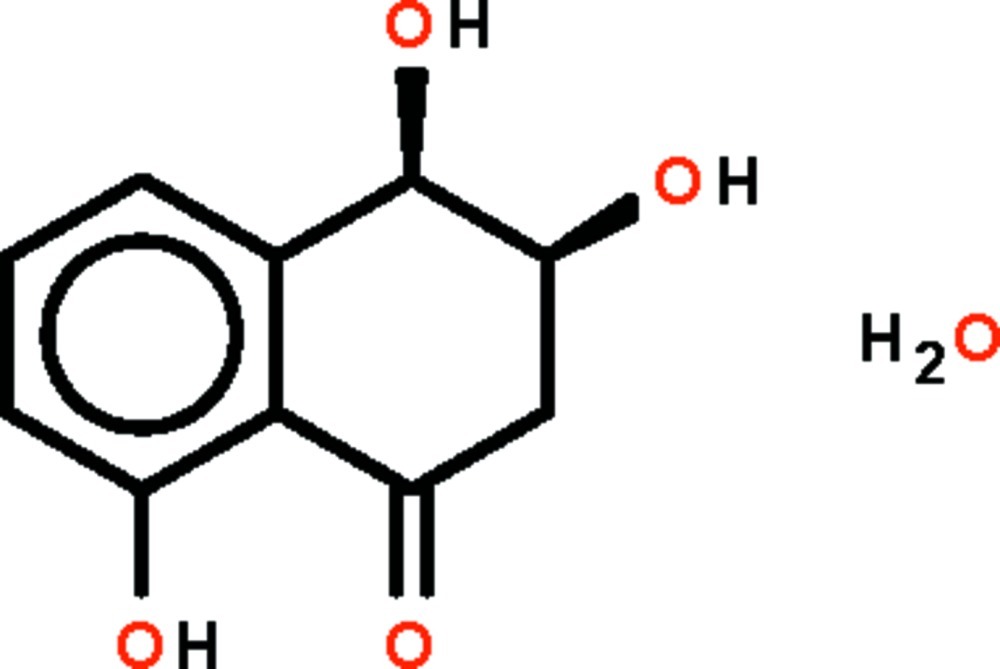


## Experimental

### 

#### Crystal data

C_10_H_10_O_4_·H_2_O
*M* *_r_* = 212.20
Orthorhombic, 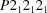

*a* = 4.6430 (4) Å
*b* = 14.3904 (11) Å
*c* = 14.4976 (10) Å
*V* = 968.65 (13) Å^3^
*Z* = 4
Mo *K*α radiation
μ = 0.12 mm^−1^
*T* = 293 K
0.31 × 0.28 × 0.24 mm

#### Data collection

Bruker APEX DUO diffractometer
6331 measured reflections
1320 independent reflections
916 reflections with *I* > 2σ(*I*)
*R*_int_ = 0.063

#### Refinement

*R*[*F* ^2^ > 2σ(*F* ^2^)] = 0.039
*wR*(*F* ^2^) = 0.096
*S* = 0.99
1320 reflections
156 parameters
5 restraints
H atoms treated by a mixture of independent and constrained refinement
Δρ_max_ = 0.21 e Å^−3^
Δρ_min_ = −0.21 e Å^−3^

### 

Data collection: *APEX2* (Bruker, 2010 ▶); cell refinement: *SAINT* (Bruker, 2010 ▶); data reduction: *SAINT*; program(s) used to solve structure: *SHELXS97* (Sheldrick, 2008 ▶); program(s) used to refine structure: *SHELXL97* (Sheldrick, 2008 ▶); molecular graphics: *X-SEED* (Barbour, 2001 ▶); software used to prepare material for publication: *publCIF* (Westrip, 2010 ▶).

## Supplementary Material

Crystal structure: contains datablock(s) global, I. DOI: 10.1107/S1600536812022623/xu5544sup1.cif

Structure factors: contains datablock(s) I. DOI: 10.1107/S1600536812022623/xu5544Isup2.hkl

Supplementary material file. DOI: 10.1107/S1600536812022623/xu5544Isup3.cml

Additional supplementary materials:  crystallographic information; 3D view; checkCIF report

## Figures and Tables

**Table 1:** Hydrogen-bond geometry (Å, °)

*D*—H⋯*A*	*D*—H	H⋯*A*	*D*⋯*A*	*D*—H⋯*A*
O1—H1⋯O2	0.84 (1)	1.87 (3)	2.590 (3)	143 (3)
O1—H1⋯O1*w*^i^	0.84 (1)	2.27 (3)	2.829 (3)	124 (3)
O3—H2⋯O1^ii^	0.84 (1)	2.15 (3)	2.924 (3)	153 (5)
O4—H3⋯O1*w*^iii^	0.85 (1)	1.82 (1)	2.657 (3)	170 (4)
O1*w*—H4⋯O2	0.84 (1)	1.98 (1)	2.805 (3)	167 (4)
O1*w*—H5⋯O4^iv^	0.85 (1)	1.88 (1)	2.726 (3)	177 (3)

Symmetry codes: (i) 
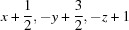
; (ii) 
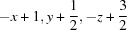
; (iii) 
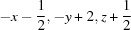
; (iv) 
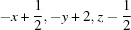
.

**Fig. 1.**
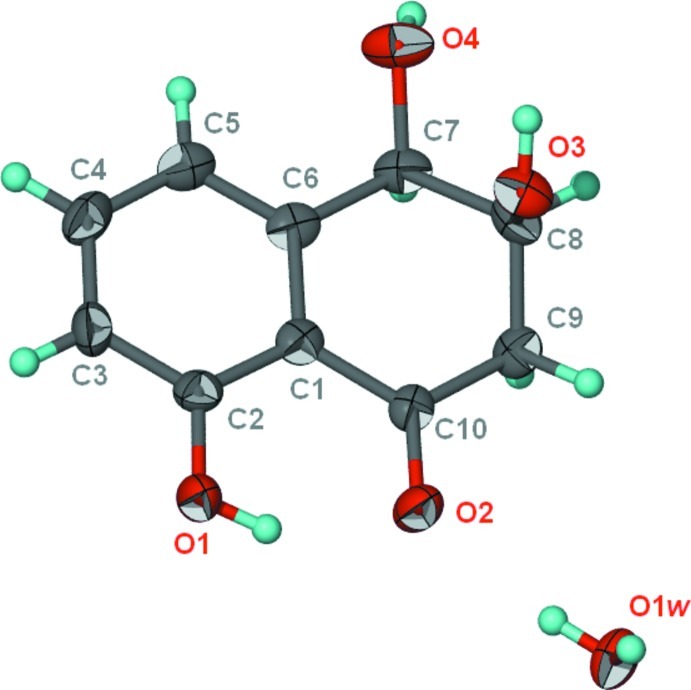
Thermal ellipsoid plot (Barbour, 2001) of C10H10O4.H2O at the 70% probability level. Hydrogen atoms are drawn as spheres of arbitrary radius.

## supplementary crystallographic information

### Comment

3,4-Dihydro-3,4,8-trihydroxy-1[*2H*]-naphthalenone is a secondary metabolite produced by several endophytic fungi,e.g., *Hypoxylon mammatum* (Borgschulte *et al.*, 1991), *Nigrospora* sp. (Trisuwan *et al.*, 2008) and *Pyrichularia orayzae* (Iwasaki *et al.*, 1972). The compound was isolated from *Embellisia eureka* in this study; the compound was found to crystallize as a monohydrate (Scheme I). In the hydrate, C_10_H_10_O_4_^.^H_2_O, the six-membered aliphatic ring that is fused to the benzene ring has a soft shape. The C-3 atom represents the sofa back. The hydroxy group of the aliphatic ring occupies a quasi-axial position (Fig. 1). The hydroxy group of the aromatic ring is hydrogen-bond donor to the carbonyl O atom; other O—H···O hydrogen bonds link the organic molecule and water molecule to form a 3D network (Table 1).

### Experimental

Fungal extraction

The fungal strain, *Embellisia eureka*, was identified by PCR. About 250 ml of ethyl acetate was added into each culture material of the fungus in an Erlenmeyer flask. The ethyl acetate phase was then concentrated under reduced pressure. The residue was diluted in 90% aqueous methanol and further extracted with *n*-hexane to remove fatty acids and other non-polar constituents. The remaining 90% methanol phase was evaporated under reduced pressure to yield 3.0 g of crude product.

Isolation protocol of 3,4-dihydro-3,4,8-trihydroxy-1[*2H*]-naphthalenone

The 90% methanol extract was submitted to vacuum liquid chromatography on a column packed with silica as the stationary phase,. The resulting fraction was submitted two successive fractionations on a Sephadex column packed with Sephadex LH-20 as stationary phase. The mobile phase was the 100% methanol. This gave 113.4 mg of a material that was purified by using the semi-preparative HPLC to give 7.0 mg of the pure compound. Crystals were obtained by slow evaporation of a methanol: water (9:1) solution of the compound.

### Refinement

The aromatic and methylene H-atoms were placed in calculated positions (C–H 0.93–0.97 Å) and were included in the refinement in the riding model approximation, with *U*(H) set to 1.2*U*(C). The hydroxy and water H-atoms were located in a difference Fourier map, and were refined with a distance restraint of 0.84±0.01 Å; their temperature factors were refined.

The (0 1 1) reflection was omitted owing to bad disagreement.

The absolute configuration was assumed from published assignments (Trisuwan *et al.*, 2008); 892 Friedel pairs were merged.

### Crystal data

**Table:**

C_10_H_10_O_4_·H_2_O	*F*(000) = 448
*M**_r_* = 212.20	*D*_x_ = 1.455 Mg m^−^^3^
Orthorhombic, *P*2_1_2_1_2_1_	Mo *K*α radiation, λ = 0.71073 Å
Hall symbol: P 2ac 2ab	Cell parameters from 1057 reflections
*a* = 4.6430 (4) Å	θ = 2.8–21.8°
*b* = 14.3904 (11) Å	µ = 0.12 mm^−^^1^
*c* = 14.4976 (10) Å	*T* = 293 K
*V* = 968.65 (13) Å^3^	Prism, brown
*Z* = 4	0.31 × 0.28 × 0.24 mm

### Data collection

**Table:**

Bruker APEX DUO diffractometer	916 reflections with *I* > 2σ(*I*)
Radiation source: fine-focus sealed tube	*R*_int_ = 0.063
Graphite monochromator	θ_max_ = 27.5°, θ_min_ = 2.8°
ω scans	*h* = −6→5
6331 measured reflections	*k* = −18→17
1320 independent reflections	*l* = −18→18

### Refinement

**Table:**

Refinement on *F*^2^	Primary atom site location: structure-invariant direct methods
Least-squares matrix: full	Secondary atom site location: difference Fourier map
*R*[*F*^2^ > 2σ(*F*^2^)] = 0.039	Hydrogen site location: inferred from neighbouring sites
*wR*(*F*^2^) = 0.096	H atoms treated by a mixture of independent and constrained refinement
*S* = 0.99	*w* = 1/[σ^2^(*F*_o_^2^) + (0.0507*P*)^2^] where *P* = (*F*_o_^2^ + 2*F*_c_^2^)/3
1320 reflections	(Δ/σ)_max_ = 0.001
156 parameters	Δρ_max_ = 0.21 e Å^−^^3^
5 restraints	Δρ_min_ = −0.21 e Å^−^^3^

### Fractional atomic coordinates and isotropic or equivalent isotropic displacement parameters (Å^2^)

**Table:**

	*x*	*y*	*z*	*U*_iso_*/*U*_eq_	
O1	0.5051 (5)	0.75199 (14)	0.74392 (14)	0.0280 (6)	
O2	0.1740 (5)	0.83227 (14)	0.62424 (12)	0.0281 (5)	
O3	0.1933 (5)	1.08380 (14)	0.69719 (15)	0.0262 (5)	
O4	−0.0651 (5)	1.10191 (16)	0.87414 (16)	0.0318 (5)	
O1w	0.0565 (5)	0.82560 (15)	0.43458 (14)	0.0268 (5)	
C1	0.1862 (7)	0.8812 (2)	0.77911 (18)	0.0198 (6)	
C2	0.3877 (7)	0.81217 (18)	0.80514 (18)	0.0226 (7)	
C3	0.4788 (7)	0.8053 (2)	0.89602 (18)	0.0270 (7)	
H3A	0.6084	0.7592	0.9134	0.032*	
C4	0.3753 (7)	0.8673 (2)	0.96024 (19)	0.0278 (8)	
H4A	0.4351	0.8622	1.0213	0.033*	
C5	0.1843 (7)	0.9371 (2)	0.9362 (2)	0.0247 (7)	
H5A	0.1202	0.9787	0.9809	0.030*	
C6	0.0881 (7)	0.94556 (19)	0.8461 (2)	0.0211 (6)	
C7	−0.1173 (7)	1.02214 (19)	0.8181 (2)	0.0244 (7)	
H7	−0.3147	1.0008	0.8293	0.029*	
C8	−0.0888 (7)	1.0480 (2)	0.7169 (2)	0.0235 (7)	
H8	−0.2352	1.0942	0.7004	0.028*	
C9	−0.1250 (6)	0.96220 (19)	0.6583 (2)	0.0236 (7)	
H9A	−0.3192	0.9385	0.6658	0.028*	
H9B	−0.0993	0.9786	0.5940	0.028*	
C10	0.0858 (6)	0.88749 (19)	0.68341 (18)	0.0193 (6)	
H1	0.439 (8)	0.761 (2)	0.6908 (13)	0.049 (12)*	
H2	0.228 (12)	1.1339 (18)	0.725 (3)	0.103 (19)*	
H3	−0.228 (4)	1.118 (3)	0.895 (2)	0.061 (13)*	
H4	0.071 (10)	0.821 (2)	0.4923 (8)	0.060 (13)*	
H5	0.212 (4)	0.848 (2)	0.414 (2)	0.039 (11)*	

### Atomic displacement parameters (Å^2^)

**Table:**

	*U*^11^	*U*^22^	*U*^33^	*U*^12^	*U*^13^	*U*^23^
O1	0.0361 (14)	0.0245 (12)	0.0236 (11)	0.0091 (10)	−0.0056 (11)	−0.0015 (10)
O2	0.0319 (12)	0.0308 (11)	0.0215 (10)	0.0076 (11)	−0.0046 (10)	−0.0056 (9)
O3	0.0207 (11)	0.0228 (12)	0.0351 (12)	−0.0052 (10)	0.0051 (10)	−0.0006 (10)
O4	0.0180 (11)	0.0354 (13)	0.0420 (13)	0.0034 (11)	0.0007 (11)	−0.0182 (11)
O1w	0.0195 (12)	0.0378 (13)	0.0231 (11)	−0.0054 (11)	−0.0032 (10)	0.0044 (10)
C1	0.0176 (15)	0.0199 (14)	0.0220 (14)	−0.0065 (12)	0.0019 (12)	0.0013 (12)
C2	0.0261 (17)	0.0187 (15)	0.0229 (14)	−0.0045 (13)	−0.0005 (13)	−0.0019 (12)
C3	0.034 (2)	0.0230 (16)	0.0243 (15)	−0.0021 (14)	−0.0067 (14)	0.0071 (14)
C4	0.034 (2)	0.0321 (17)	0.0175 (14)	−0.0094 (15)	−0.0003 (14)	0.0024 (13)
C5	0.0224 (16)	0.0282 (17)	0.0235 (15)	−0.0068 (14)	0.0066 (14)	−0.0030 (13)
C6	0.0153 (14)	0.0246 (15)	0.0234 (15)	−0.0070 (13)	0.0052 (13)	−0.0015 (12)
C7	0.0173 (16)	0.0263 (16)	0.0298 (16)	−0.0011 (13)	0.0007 (14)	−0.0069 (14)
C8	0.0157 (14)	0.0209 (15)	0.0340 (16)	0.0029 (13)	0.0003 (13)	0.0015 (13)
C9	0.0158 (16)	0.0299 (16)	0.0252 (16)	0.0005 (13)	−0.0031 (13)	−0.0006 (13)
C10	0.0155 (14)	0.0215 (14)	0.0211 (13)	−0.0048 (13)	−0.0004 (13)	0.0016 (12)

### Geometric parameters (Å, º)

**Table:**

O1—C2	1.355 (3)	C3—H3A	0.9300
O1—H1	0.840 (10)	C4—C5	1.384 (4)
O2—C10	1.239 (3)	C4—H4A	0.9300
O3—C8	1.436 (4)	C5—C6	1.386 (4)
O3—H2	0.841 (10)	C5—H5A	0.9300
O4—C7	1.427 (3)	C6—C7	1.513 (4)
O4—H3	0.846 (10)	C7—C8	1.520 (4)
O1w—H4	0.842 (10)	C7—H7	0.9800
O1w—H5	0.846 (10)	C8—C9	1.508 (4)
C1—C6	1.417 (4)	C8—H8	0.9800
C1—C2	1.416 (4)	C9—C10	1.498 (4)
C1—C10	1.466 (4)	C9—H9A	0.9700
C2—C3	1.387 (4)	C9—H9B	0.9700
C3—C4	1.377 (4)		
			
C2—O1—H1	111 (3)	O4—C7—C6	109.1 (2)
C8—O3—H2	113 (4)	O4—C7—C8	109.7 (2)
C7—O4—H3	106 (3)	C6—C7—C8	112.5 (3)
H4—O1w—H5	109 (4)	O4—C7—H7	108.5
C6—C1—C2	119.2 (2)	C6—C7—H7	108.5
C6—C1—C10	120.4 (3)	C8—C7—H7	108.5
C2—C1—C10	120.3 (2)	O3—C8—C9	106.4 (2)
O1—C2—C3	117.0 (3)	O3—C8—C7	111.0 (2)
O1—C2—C1	122.7 (2)	C9—C8—C7	109.5 (2)
C3—C2—C1	120.3 (3)	O3—C8—H8	109.9
C4—C3—C2	119.3 (3)	C9—C8—H8	109.9
C4—C3—H3A	120.3	C7—C8—H8	109.9
C2—C3—H3A	120.3	C10—C9—C8	112.2 (2)
C3—C4—C5	121.6 (3)	C10—C9—H9A	109.2
C3—C4—H4A	119.2	C8—C9—H9A	109.2
C5—C4—H4A	119.2	C10—C9—H9B	109.2
C4—C5—C6	120.5 (3)	C8—C9—H9B	109.2
C4—C5—H5A	119.8	H9A—C9—H9B	107.9
C6—C5—H5A	119.8	O2—C10—C1	120.7 (3)
C5—C6—C1	119.0 (3)	O2—C10—C9	120.5 (2)
C5—C6—C7	121.3 (3)	C1—C10—C9	118.8 (3)
C1—C6—C7	119.6 (3)		
			
C6—C1—C2—O1	−175.8 (3)	C1—C6—C7—O4	−148.4 (3)
C10—C1—C2—O1	2.8 (4)	C5—C6—C7—C8	153.2 (3)
C6—C1—C2—C3	2.6 (4)	C1—C6—C7—C8	−26.4 (4)
C10—C1—C2—C3	−178.9 (3)	O4—C7—C8—O3	59.1 (3)
O1—C2—C3—C4	177.3 (3)	C6—C7—C8—O3	−62.5 (3)
C1—C2—C3—C4	−1.2 (4)	O4—C7—C8—C9	176.3 (2)
C2—C3—C4—C5	−0.6 (5)	C6—C7—C8—C9	54.7 (3)
C3—C4—C5—C6	1.0 (5)	O3—C8—C9—C10	63.4 (3)
C4—C5—C6—C1	0.5 (4)	C7—C8—C9—C10	−56.7 (3)
C4—C5—C6—C7	−179.1 (3)	C6—C1—C10—O2	178.9 (3)
C2—C1—C6—C5	−2.2 (4)	C2—C1—C10—O2	0.4 (4)
C10—C1—C6—C5	179.2 (3)	C6—C1—C10—C9	−0.8 (4)
C2—C1—C6—C7	177.4 (3)	C2—C1—C10—C9	−179.3 (3)
C10—C1—C6—C7	−1.1 (4)	C8—C9—C10—O2	−149.4 (3)
C5—C6—C7—O4	31.2 (4)	C8—C9—C10—C1	30.4 (3)

### Hydrogen-bond geometry (Å, º)

**Table:**

*D*—H···*A*	*D*—H	H···*A*	*D*···*A*	*D*—H···*A*
O1—H1···O2	0.84 (1)	1.87 (3)	2.590 (3)	143 (3)
O1—H1···O1*w*^i^	0.84 (1)	2.27 (3)	2.829 (3)	124 (3)
O3—H2···O1^ii^	0.84 (1)	2.15 (3)	2.924 (3)	153 (5)
O4—H3···O1*w*^iii^	0.85 (1)	1.82 (1)	2.657 (3)	170 (4)
O1*w*—H4···O2	0.84 (1)	1.98 (1)	2.805 (3)	167 (4)
O1*w*—H5···O4^iv^	0.85 (1)	1.88 (1)	2.726 (3)	177 (3)

Symmetry codes: (i) *x*+1/2, −*y*+3/2, −*z*+1; (ii) −*x*+1, *y*+1/2, −*z*+3/2; (iii) −*x*−1/2, −*y*+2, *z*+1/2; (iv) −*x*+1/2, −*y*+2, *z*−1/2.
